# Cerebrospinal fluid neurogranin concentration in neurodegeneration: relation to clinical phenotypes and neuropathology

**DOI:** 10.1007/s00401-018-1851-x

**Published:** 2018-04-26

**Authors:** Erik Portelius, Bob Olsson, Kina Höglund, Nicholas C. Cullen, Hlin Kvartsberg, Ulf Andreasson, Henrik Zetterberg, Åsa Sandelius, Leslie M. Shaw, Virginia M. Y. Lee, David J. Irwin, Murray Grossman, Daniel Weintraub, Alice Chen-Plotkin, David A. Wolk, Leo McCluskey, Lauren Elman, Jennifer McBride, Jon B. Toledo, John Q. Trojanowski, Kaj Blennow

**Affiliations:** 1 Department of Psychiatry and Neurochemistry, Institute of Neuroscience and Physiology, The Sahlgrenska Academy at the University of Gothenburg, 431 80, Mölndal, Sweden; 2 Clinical Neurochemistry Laboratory, Sahlgrenska University Hospital, Mölndal, Sweden; 3 Department of Molecular Neuroscience, UCL Institute of Neurology, Queen Square, London, WC1E 6BT UK; 4 UK Dementia Research Institute, London, WC1E 6BT UK; 5 Department of Pathology and Laboratory Medicine, Institute on Aging, Center for Neurodegenerative Disease Research, University of Pennsylvania School of Medicine, Philadelphia, PA USA; 6 Department of Neurology, University of Pennsylvania School of Medicine, Philadelphia, PA USA; 7 Department of Psychiatry, University of Pennsylvania School of Medicine, Philadelphia, PA USA; 8 Parkinson’s Disease and Mental Illness Research, Education and Clinical Centers (PADRECC and MIRECC), Philadelphia Veterans Affairs Medical Center, Philadelphia, PA USA; 9 Department of Neurology, Houston Methodist Hospital, Houston, TX USA

**Keywords:** Alzheimer’s disease, Neurogranin, Biomarker, Neuropathology, Cerebrospinal fluid

## Abstract

Neurogranin (Ng) is a post-synaptic protein that previously has been shown to be a biomarker for synaptic function when measured in cerebrospinal fluid (CSF). The CSF concentration of Ng is increased in Alzheimer’s disease dementia (ADD), and even in the pre-dementia stage. In this prospective study, we used an enzyme-linked immunosorbent assay that quantifies Ng in CSF to test the performance of Ng as a marker of synaptic function. In 915 patients, CSF Ng was evaluated across several different neurodegenerative diseases. Of these 915 patients, 116 had a neuropathologically confirmed definitive diagnosis and the relation between CSF Ng and topographical distribution of different pathologies in the brain was evaluated. CSF Ng was specifically increased in ADD compared to eight other neurodegenerative diseases, including Parkinson’s disease (*p* < 0.0001), frontotemporal dementia (*p* < 0.0001), and amyotrophic lateral sclerosis (*p* = 0.0002). Similar results were obtained in neuropathologically confirmed cases. Using a biomarker index to evaluate whether CSF Ng contributed diagnostic information to the core AD CSF biomarkers (amyloid β (Aβ), t-tau, and p-tau), we show that Ng significantly increased the discrimination between AD and several other disorders. Higher CSF Ng levels were positively associated with greater Aβ neuritic plaque (Consortium to Establish a Registry for Alzheimer’s Disease (CERAD) neuritic plaque score, *p* = 0.0002) and tau tangle pathology (Braak neurofibrillary tangles staging, *p* = 0.0007) scores. In the hippocampus and amygdala, two brain regions heavily affected in ADD with high expression of Ng, CSF Ng was associated with plaque (*p* = 0.0006 and *p* < 0.0001), but not with tangle, α-synuclein, or TAR DNA-binding protein 43 loads. These data support that CSF Ng is increased specifically in ADD, that high CSF Ng concentrations likely reflect synaptic dysfunction and that CSF Ng is associated with β-amyloid plaque pathology.

**Electronic supplementary material:**

The online version of this article (10.1007/s00401-018-1851-x) contains supplementary material, which is available to authorized users.

## Introduction

Synaptic density, and thus synaptic protein expression, is highest in the associative cortical areas, probably reflecting cognitive processing [4]. In Alzheimer’s disease dementia (ADD) these brain regions show synaptic dysfunction, degeneration, and loss; synaptic pathology occurs early in the disease process, perhaps even earlier than neuronal degeneration and loss [2, 10], which is supported by studies of a tauopathy mouse model [56]. In addition, neuropathological studies have shown that this synaptic dysfunction is more linked to memory dysfunction than plaque and tangle pathologies, which are the two major pathological hallmarks of ADD [3, 9, 29, 48].

Neurogranin (Ng) is a neuronal protein that is highly expressed in the cortex, hippocampus, and amygdala, with the highest concentrations at the dendritic spines [16, 42]. Since the discovery that Ng is present in CSF [8], and that levels are increased in ADD [50], several recent studies have reported higher cerebrospinal fluid (CSF) Ng concentration in ADD and mild cognitive impairment (MCI) patients compared to cognitively unimpaired elderly subjects [26–28, 43]. Further, other studies suggest that increased CSF Ng concentrations may be specific for ADD [25, 41, 53].

Recently, we showed that CSF Ng concentrations can predict the rate of cognitive decline in prodromal ADD and conversion from MCI to ADD [39]. Accordingly, CSF Ng seems to be a novel biomarker reflecting ADD-associated synaptic dysfunction, which may be used to improve early diagnosis and prognostication, as well as monitoring effects of disease-modifying drug candidates on synaptic status.

In CSF, Ng is present as several endogenous peptides of different lengths, as well as full-length protein (78 amino acids) [27]. To further explore CSF Ng as a biomarker for ADD, we optimized and validated an enzyme-linked immunosorbent assay (ELISA) that quantifies C-terminal Ng peptides and full-length Ng protein in CSF. Here we present results on CSF Ng in a very large clinical cohort including several different neurodegenerative diseases with a subset followed to autopsy for determination of the neuropathology definitive diagnoses. The specific hypotheses tested were that increased CSF Ng is specific to ADD, and that patients with autopsy-confirmed ADD have higher CSF Ng concentrations compared to patients with dementia with Lewy bodies (DLB), frontotemporal dementia (FTD), progressive supranuclear palsy (PSP) or amyotrophic lateral sclerosis (ALS). Last, we wanted to explore the relationship between CSF Ng concentrations and the topographical distribution of neuritic plaques and tau tangles in the brain.

## Materials and methods

### Subjects

Subjects included 75 controls (CTRL), 114 MCI, 397 ADD, 6 posterior cortical atrophy (PCA), 96 FTD [46 behavioral variant FTD (bvFTD), 12 logopenic variant primary progressive aphasia (lvPPA), 20 non-fluent variant primary progressive aphasia (nfvPPA), 18 semantic variant PPA (svPPA)], 68 ALS, 37 Parkinson’s disease with normal cognition (PD), 19 PD with MCI (PD MCI), 29 PD with dementia (PDD), 33 DLB, 21 corticobasal syndrome (CBS), and 20 PSP patients. Recruitment of the patients and diagnostic criteria for the groups have been described previously in detail [20, 52, 55]. Demographic and biomarker characteristics of the patients included in the study are shown in Table 1. Patients were clinically evaluated at each clinical core [52] and current clinical criteria were used for diagnosis of AD [31, 32], bvFTD [40], PPA [15], CBS [1], PSP [17], ALS [46] and DLB [30]. The onset of disease was defined by the year reported by patients/family of functional impairment in cognitive/motor features. Standardized neuropsychological assessments were collected by trained examiners at each center. All patients were evaluated at the clinical cores at the University Of Pennsylvania Perelman School Of Medicine including the Penn Alzheimer’s disease core center, Frontotemporal degeneration center, Udall Center for Parkinson’s’ disease research and Amyotrophic lateral sclerosis center. CSF samples were collected in a standardized manner using the standard operating procedures of ADNI as described (http://www.adni-info.org/). Out of the 915 subjects included in the study, 116 had a definitive diagnosis by neuropathology. The definitive diagnostic groups included ADD (*n* = 75), DLB (*n* = 16), FTD (*n* = 12), ALS (*n* = 7), and PSP (*n* = 6) subjects. The neuropathological data and criteria have been described elsewhere [19, 37, 51, 52]. See Online Resource 1 for demographics.

**Table 1:** Demographic and clinical characteristics of subjects included in the study

	CTRL	MCI	ADD	PDMCI	PD	PDD	PCA	DLB	CBS	PSP	ALS	bvFTD	lvPPA	nfvPPA	svPPA
*n*	75	114	397	19	37	29	6	33	21	20	68	46	12	20	18
Gender, *n*, female/male	50/25	58/56	236/161	3/16	11/26	4/25	5/1	14/19	12/9	10/10	18/50	13/33	7/5	9/11	11/7
Age at LP, years	69 [61–75]	73 [66–78]	73 [66–77]	65 [62–68]	65 [62–71]	74 [66–79]	61 [54–95]	71 [67–80]	63 [62–72]	71 [60–73]	60 [53–68]***	61 [55–67]**	62 [59–68]	65 [59–71]	64 [59–69]
First MMSE	29 [29, 30]	27 [24–28]***	23 [18–26]***	28 [27–30]	29 [28–30]	27 [25–29]*	23 [18–28]*	22 [19–25]***	26 [20–28]**	28 [25–30]	28 [25–30]	27 [25–28]*	24 [18–28]**	22 [16–27]***	24 [18–27]***
*APOE* genotype (*n*)															
*E2/E2*	1		1		1						1				
*E2/E3*	7	5	15	2	4	3		2	3	2	5	4			1
*E2/E4*		2	6	2	1				1	1	2	1	4	1	
*E3/E3*	41	51	115	10	23	14	3	14	12	16	43	25	7	8	14
*E3/E4*	21	32	148	3	6	12	2	9	3	1	13	11		9	2
* E4/E4*	1	6	59		1			2	1		1	2		2	1
Duration of disease	N/A	3 [1–4]	3 [2–4]	6 [4–14]	7 [4–10]	10 [4–15]	3 [1–6]	3 [2–4]	3 [2–4]	4 [2–5]	1 [1–2]	3 [2–5]	3 [1–5]	3 [2–5]	3 [2–6]
CSF Aβ_42_, pg/mL	244 [212–298]	178 [126–230]***	134 [107–160]***	232 [202–288]	274 [219–306]	214 [161–260]	260 [125–308]	147 [127–172]***	225 [166–293]	210 [167–260]	267 [213–323]	238 [174–313]	139 [121–153]**	193 [172–280]	282 [178–309]
CSF t-tau, pg/mL	50 [38–68]	63 [42–100]	104 [75–155]***	39 [32–56]	40 [36–55]	48 [36–67]	53 [42–123]	54 [35–89]	71 [62–108]	50 [36–75]	50 [36–70]	56 [41–75]	130 [89–157]**	56 [42–87]	89 [59–119]
CSF p-tau, pg/mL	17 [12–23]	22 [13–41]	36 [23–58]***	18 [13–35]	17 [13–28]	22 [12–26]	26 [11–37]	16 [12–28]	21 [16–28]	12 [10–19]	11 [8–15]*	16 [12–23]	39 [26–47]*	16 [12–29]	15 [12–28]
CSF Ng, pg/mL	133 [87–189]	130 [59–267]	203 [115–338]*	102 [79–181]	117 [58–199]	91 [64–208]	116 [73–194]	126 [58–260]	157 [89–231]	104 [27–244]	86 [42–135]*	81 [35–126]*	204 [118–287]	104 [48–215]	160 [75–212]

The values presented are medians [interquartile range]

*p < 0.05 vs controls, **p < 0.001 vs controls, ***p < 0.0001 vs controls

Genomic DNA was extracted from peripheral blood before death or frozen brain samples postmortem as described elsewhere [23]. *APOE* allele status was defined using two SNPs (rs7412 and rs429358) which were genotyped by TaqMan allelic discrimination assays (Thermo-Fisher, USA).

Braak tau neurofibrillary tangle staging (PHF-1) and the Consortium to Establish a Registry for Alzheimer’s Disease (CERAD) neuritic plaque score (thioflavin stain) were used to classify ADD neuropathology into four groups as described previously [19, 21]: no (or negligible) ADD neuropathology (0), low-level ADD (1), intermediate-level ADD (2), and high-level ADD (3). For the determination of neuronal loss, the brain sections were stained with hematoxylin–eosin and Thal staging (nab228) (*n* = 114) was performed as described elsewhere [52]. An ABC score that incorporates histopathologic assessments of Aβ deposits (A), staging of neurofibrillary tangles (B), and scoring of neuritic plaques (C) was calculated as described [37].

Postmortem examination and scoring of tau, Aβ, α-synuclein, and TAR DNA-binding protein 43 (TDP-43) pathology were performed on amygdala and cornu ammonis/subiculum-hippocampus as described elsewhere [52].

The Alzheimer’s Disease Core Center (ADCC), Penn Memory Center, the Frontotemporal Degeneration Center, the ALS Center, the Parkinson’s Disease and Movement Disorder Clinic, and the Penn Udall Center for Parkinson’s Research each have protocols approved by the institutional review board to recruit patients, along with their clinical data, into research studies. In addition, these centers invite patients to participate in the brain donation program.

### CSF measurements

The generation and purification of the anti-Ng monoclonal antibodies (Mab) NG22 (epitope 63–75) and NG2 (epitope 52–63) were performed as described previously [44]. 96-well plates were coated with 3 µg/mL (100 µL/well) of the Mab NG22 in 50 mM bicarbonate buffer (pH 9.6) and incubated overnight (16–18 h) at + 4 °C. After washing four times with 0.05% Tween 20 in PBS (PBS–Tween) (350 µL/well), the remaining protein binding sites were blocked with 1% bovine serum albumin (BSA) in PBS (0.01 M phosphate buffer, 0.14 M NaCl, pH 7.4) for 1 h at + 20 °C (250 μL/well). Coated plates were then stored at − 20 °C. Prior to ELISA measurement, the plates were thawed and then washed with PBS–0.05% Tween four times (350 μL per well and wash), followed by addition of 100 µL of samples, controls, and calibrators to the plate. Recombinant full-length Ng protein with a GST-tag was used as calibrator. The calibration curve ranged from 25.9 to 3310 pg/mL (1:2 dilutions in 0.5% octyl beta-d-glucopyranoside, 1% BSA in PBS). The samples, blanks, and calibrators were incubated overnight at + 4 °C. Next day the plates were washed and then incubated for 1 h at 350 rpm (room temperature) with the detector antibody, biotinylated NG2 (2.7 µg/mL) in 1% BSA in PBS–Tween (100 µL/well). After another washing step, the plates were incubated for 30 min (room temperature) with 100 µL/well of enhanced streptavidin–HRP (Kem En Tech #4740 N) diluted 1:20 000 in 1% BSA in PBS–Tween. The plates were then washed and color reaction was started using 100 µL/well of substrate (TMB one, ready to use, Kem En Tech #4380A). After 20 min in dark the reaction was stopped using 100 µL/well of 0.2 M of H_2_SO_4_ and the absorbance was measured at 450 nm (reference wavelength 650 nm) using an ELISA plate reader (*V*_max_, Molecular Devices, USA). A fitted four-parameter logistic model was used as the calibration curve (SoftMax Pro v. 4.0, Molecular Devices, USA). The analyses were performed by board-certified laboratory technicians blinded to clinical information.

CSF collection, processing, and storage procedures have been described previously [45]. CSF Aβ42, total-tau (t-tau), and phosphorylated tau (p-tau) were measured using the multiplex xMAP Luminex platform (Luminex Corp, Austin, TX, USA) with the INNOBIA AlzBio3 kit (Innogenetics, Ghent, Belgium) as described previously [45]. Subjects were classified as ADD biomarker positive or negative using previously established cutoffs (CSF Aβ_42_ < 192 pg/mL, CSF t-tau > 93 pg/mL) that maximized the separation of autopsy-confirmed ADD cases with Aβ pathology from controls without Aβ pathology as described by Shaw et al. [45].

### Statistical analysis

Statistical analyses were performed using GraphPad Prism 7 and the R programming language (version 3.4.3), while the biomarker index model was developed using Python 3.6. Because biomarker values were skewed, non-parametric tests were used. Differences between groups were assessed using the non-parametric Kruskal–Wallis test followed by Dunn’s multiple-comparison test if significant. Because post hoc analysis involved a large number of comparisons, reported *p* values were adjusted using Holm–Bonferroni procedure to control the family-wise error rate. The associations of Ng with the other CSF biomarkers Aβ42, t-tau and p-tau were investigated with Spearman’s rank correlation (rho_s_). All tests were two sided and significance threshold was set at *p* < 0.05. We investigated the relationship between CSF Ng and MMSE change per year while adjusting for age, sex, and disease duration using partial Spearman’s rank correlation. We tested this relationship in the whole study population, only the MCI group, and only the ADD group. Additionally, a biomarker index model was developed with the goal of accurately discriminating between two disease groups on the basis of their CSF biomarker measurements. The baseline model included CSF Aβ42, CSF t-tau and CSF p-tau, and the Ng model included CSF Ng in addition to the three aforementioned CSF measurements. The discriminator itself is a support vector machine (SVM), see Online Resource 2 for a detailed description.

## Results

### CSF Ng in AD and other diagnoses

**Fig. 1:**
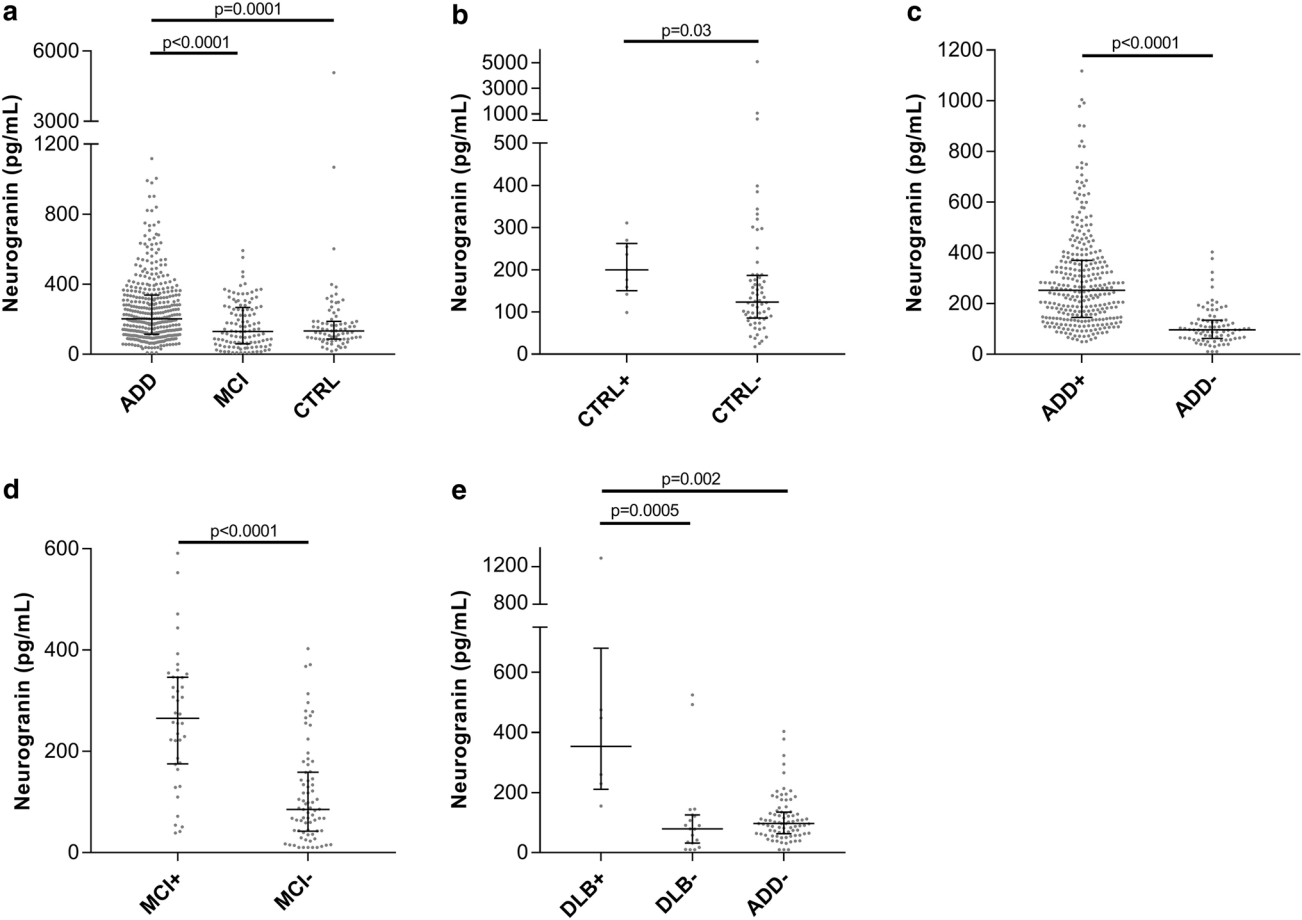
**a** Scatterplots displaying the CSF Ng concentrations in ADD, MCI and CTRL. The CTRL (**b**), ADD (**c**) MCI (**d**) and DLB (**e**) groups were divided into biomarker positive (+) or negative (−) for ADD based on previously established cutoff concentrations for t-tau and Aβ42 [45]. The bars presented in the figures are medians with interquartile ranges and comparisons between groups were performed using Kruskal–Wallis test, followed by the Mann–Whitney *U* test

CSF Ng concentrations were significantly higher in ADD compared to both MCI (*p* < 0.0001) and CTRL (*p* = 0.0001) while the concentrations were similar in CTRL and MCI (Fig. 1a). Based on previously defined cutoff concentrations for t-tau (93 pg/mL) and Aβ1-42 (192 pg/mL) [45], subjects were classified as AD biomarker positive or negative by calculating a ratio between the two biomarkers (t-tau/Aβ1-42). A value of < 0.48 was considered as AD biomarker negative. When applying this cutoff on the CTRL group, the AD biomarker-positive CTRL subjects (*n* = 9) had significantly higher CSF Ng concentrations than the AD biomarker-negative CTRL group subjects (*n* = 64, *p* = 0.03) (Fig. 1b). Subjects in the former group (*n* = 9) were excluded from the control group, as they were considered having preclinical AD pathology. Similarly, the AD biomarker-positive ADD and MCI groups both had significantly increased CSF Ng concentrations compared to the biomarker-negative group (*p* < 0.0001 for both) (Fig. 1c, d). In addition, AD biomarker-positive DLB patients had significantly increased Ng concentrations compared to biomarker-negative DLB and ADD subjects (*p* = 0.0005 and *p* = 0.002, respectively) (Fig. 1e). The ADD and MCI biomarker-negative patients were excluded from further analysis (*n* = 83 and *n* = 74 for ADD and MCI, respectively).

**Fig. 2:**
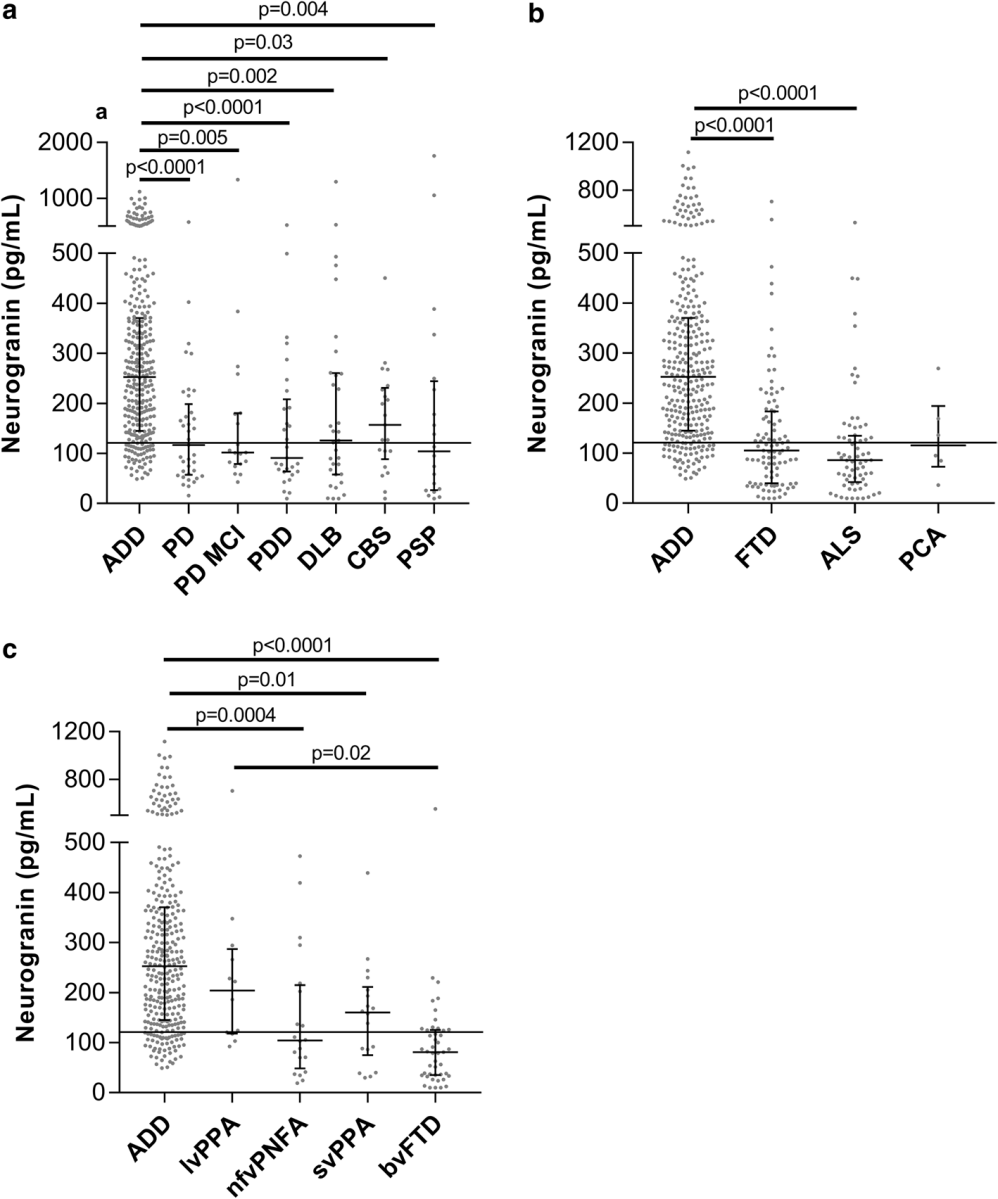
Scatterplots displaying the CSF Ng concentrations in **a** biomarker-positive ADD, PD, PD MCI, PDD, DLB CBD and PSP and **b** biomarker-positive ADD, FTD and ALS. **c** Scatterplots showing the CSF Ng concentrations after that the FTD group was divided into the subgroups PPA log, PPA PNFA, PPA SD and bvFTD. The bars presented in the figures are medians with interquartile ranges and comparisons between groups were performed using Kruskal–Wallis test, followed by the Mann–Whitney *U* test. The dashed lines represent the median for biomarker-negative CTRL

Relative to ADD, the Ng concentrations were significantly lower in the PD (*p* < 0.0001), PD MCI (*p* = 0.005), PDD (*p* < 0.0001), DLB (*p* = 0.002), CBS (*p* = 0.03), and PSP (*p* = 0.004) (Fig. 2a). There were no significant differences in CSF Ng concentrations between the atypical parkinsonian diagnosis (CBS and PSP), DLB, and PD. The ADD group had significantly higher Ng concentrations compared to both FTD (*p* < 0.0001) and ALS (*p* < 0.0001) (Fig. 2b). FTD is a pathologically heterogeneous entity that includes several related disorders in which progressive degeneration of the frontal and temporal lobes is common [15, 40]. On the basis of clinical phenotypes, we divided the FTD group into the following subgroups; bvFTD and PPA of which the latter can be further divided into nfvPPA, lvPPA, and svPPA [15]. Compared to ADD, nfvPPA, svPPA, and bvFTD had significantly lower CSF Ng concentrations (*p* = 0.0004, *p* = 0.01, and *p* < 0.0001) (Fig. 2c). Interestingly, lvPPA had significantly increased concentrations compared to bvFTD (*p* < 0.02) and similar CSF Ng concentrations as ADD (Fig. 2c). In contrast, bvFTD had a tendency towards decreased CSF Ng concentrations compared to CTRL but this did not reach statistical significance (Fig. 2c).

To test if CSF Ng contributed additional information in the context of discriminating between neurodegenerative disorders we developed a biomarker index model with the goal of accurately discriminating between two disease groups on the basis of their CSF biomarker measurements (Aβ, t-tau, p-tau and Ng). We found that Ng significantly increased the SVM model’s ability to discriminate between numerous pairs of disorders. More specifically, the inclusion of Ng led to an increase in 24% accuracy in distinguishing between CTRL and bvFTD, 3.6% increase for MCI vs bvFTD, and 9.5% increase for PD vs bvFTD. Including Ng also led to a 7.7% increase in accuracy in distinguishing between MCI and PD, 6.4% increase for MCI vs PD MCI, and 4.3% increase for MCI vs PDD (see Online Resource 3).

### CSF Ng in relation to tau and Aβ

**Fig. 3:**
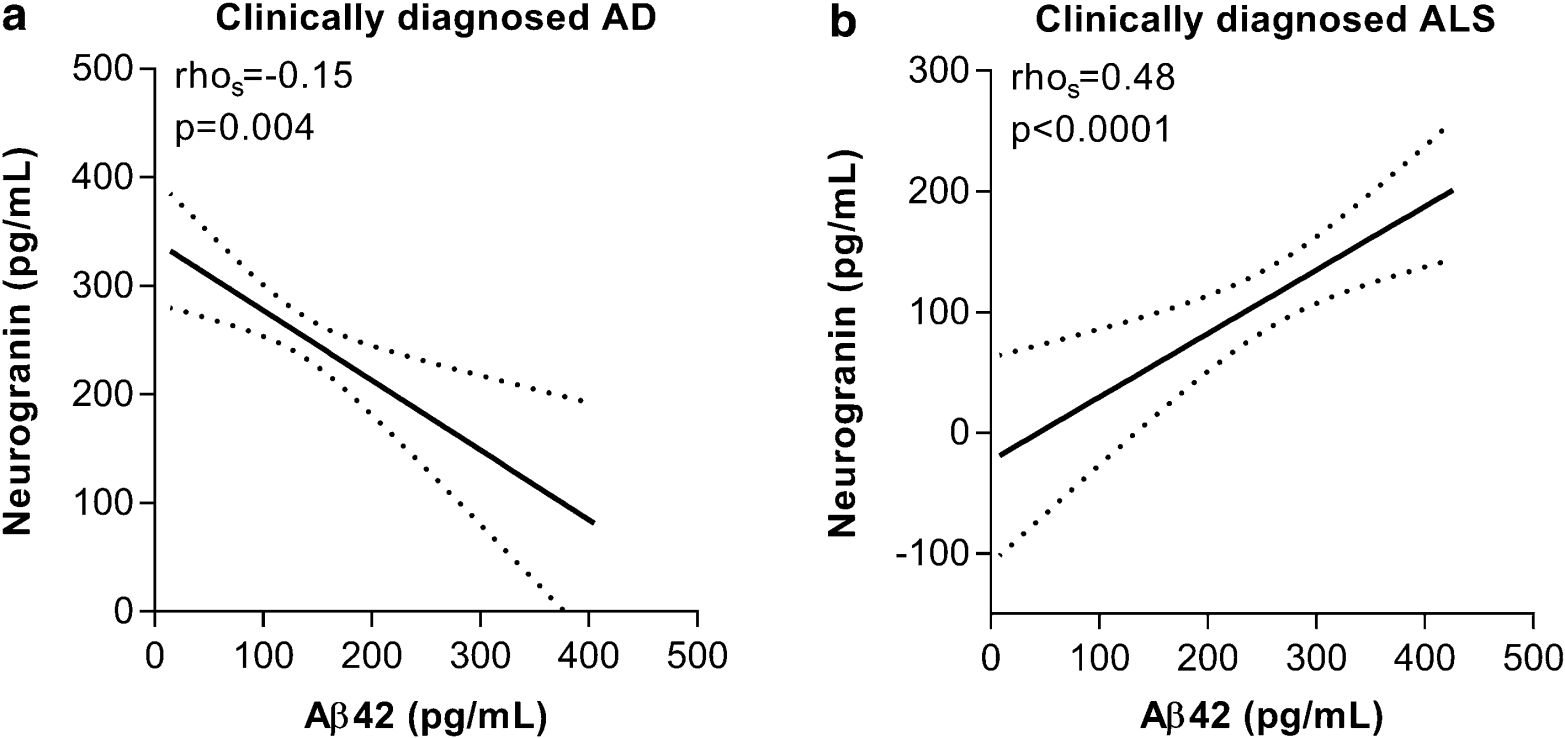
Correlations between CSF Ng concentrations and Aβ42 in clinically diagnosed ADD (**a**) and clinically diagnosed ALS (**b**) patients. The dashed lines represent the 95% confidence bands of the best-fit line. The associations were investigated with Spearman’s rank correlation

t-tau correlated positively with CSF Ng in all diagnostic groups except in PSP, PCA and lvPPA (see Table 2 for rho_s_ and *p* values). p-tau correlated with CSF Ng in CTRL (rho_s_ = 0.39), MCI (rho_s_ = 0.60), and ADD (rho_s_ = 0.56) (*p* < 0.001 for all groups) as well in PD MCI (rho_s_ = 0.52, *p* < 0.05), DLB (rho_s_ = 0.56, *p* < 0.01), and bvFTD (rho_s_ = 0.49, *p* < 0.01) but not in the other diagnoses (Table 2). CSF Ng concentration showed a weak negative correlation with CSF Aβ42 in ADD (rho_s_ = −0.15, *p* = 0.004) while a stronger positive correlation with CSF Aβ42 was found in ALS (rho_s_ = 0.48, *p* < 0.0001) (Fig. 3a, b). There were no correlations between CSF Ng and Aβ42 in the other groups.

**Table 2:** Correlations between CSF Ng and tau

Clinical diagnosis	t-tau	p-tau
CTRL	0.61***	0.39***
MCI	0.79***	0.60***
ADD	0.77***	0.56***
PD MCI	0.51*	0.52*
PD	0.75***	0.14
PDD	0.48**	0.18
PCA	0.3	− 0.3
DLB	0.77***	0.56**
CBS	0.87***	0.2
PSP	0.37	− 0.26
ALS	0.48***	− 0.097
bvFTD	0.57***	0.49**
lvPPA	0.64	0.33
nfvPPA	0.76**	0.19
svPPA	0.80**	0.35

The values are Spearman’s rank correlation coefficient

*p < 0.05, **p < 0.01, ***p < 0.001

### CSF Ng in relation to MMSE

**Fig. 4:**
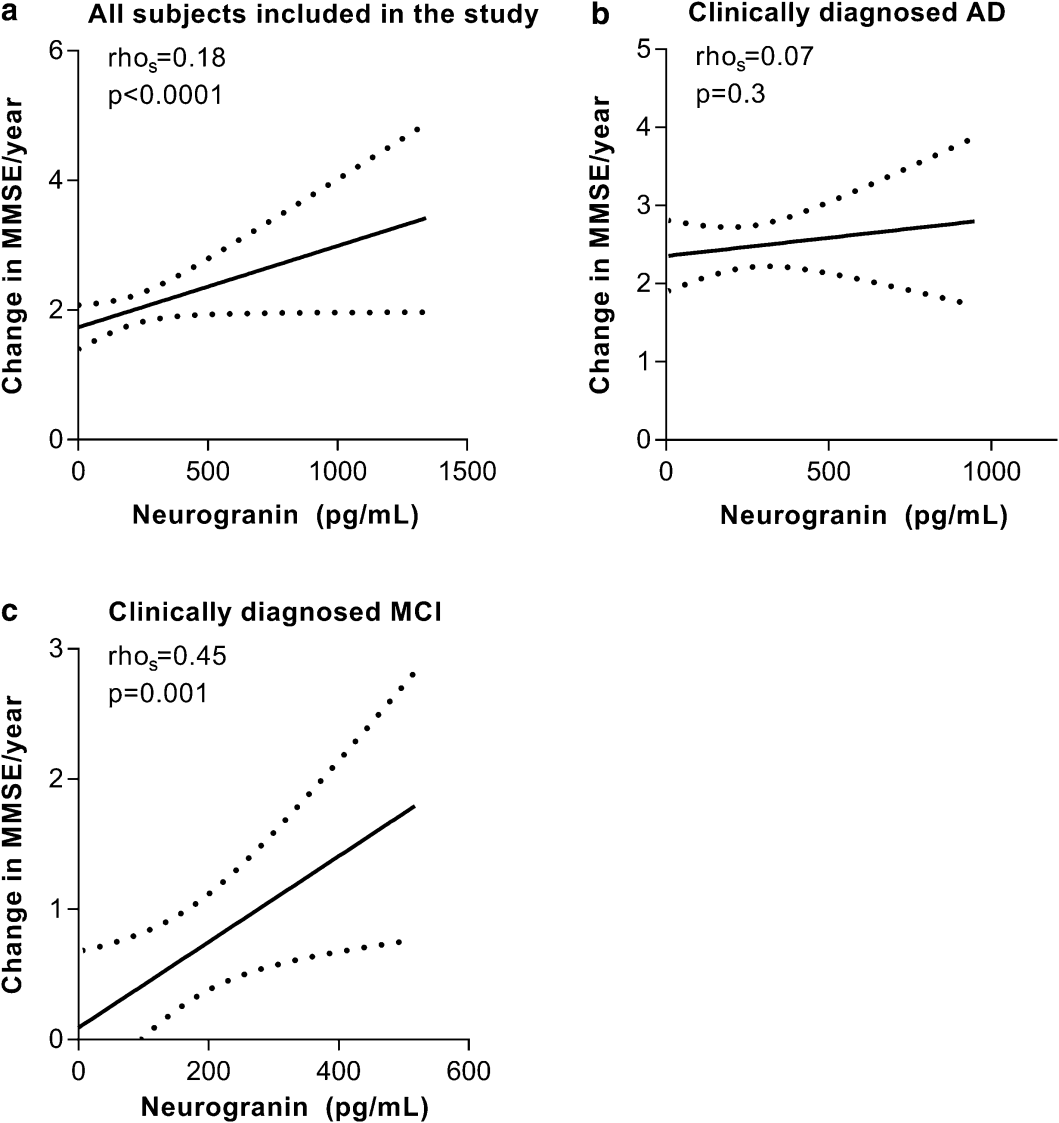
Correlations between CSF Ng concentrations and loss in MMSE points/year in **a** all subjects included in the study, **b** clinically diagnosed ADD and **c** clinically diagnosed MCI. The dashed lines represent the 95% confidence bands of the best-fit line. The associations were investigated with Spearman’s rank correlation

While controlling for age, gender, and disease duration, we found a significant association between CSF Ng concentration and longitudinal decline in MMSE scores per year in the whole study population (rho_s_ = 0.17, *p* = 0.001) (Fig. 4a). In the ADD group there was a suggestive relationship without covariate adjustment (rho_s_ = 0.12, *p* = 0.085) which was no longer observed after covariate adjustment (rho_s_ = 0.07, *p* = 0.3) (Fig. 4b). There was a significant relationship in the MCI group (rho_s_ = 0.45, *p* = 0.001) (Fig. 4c). CSF Ng concentrations did not correlate with rates of change in MMSE in the FTD, ALS, or PD groups.

### CSF Ng in relation to APOE genotype

**Fig. 5:**
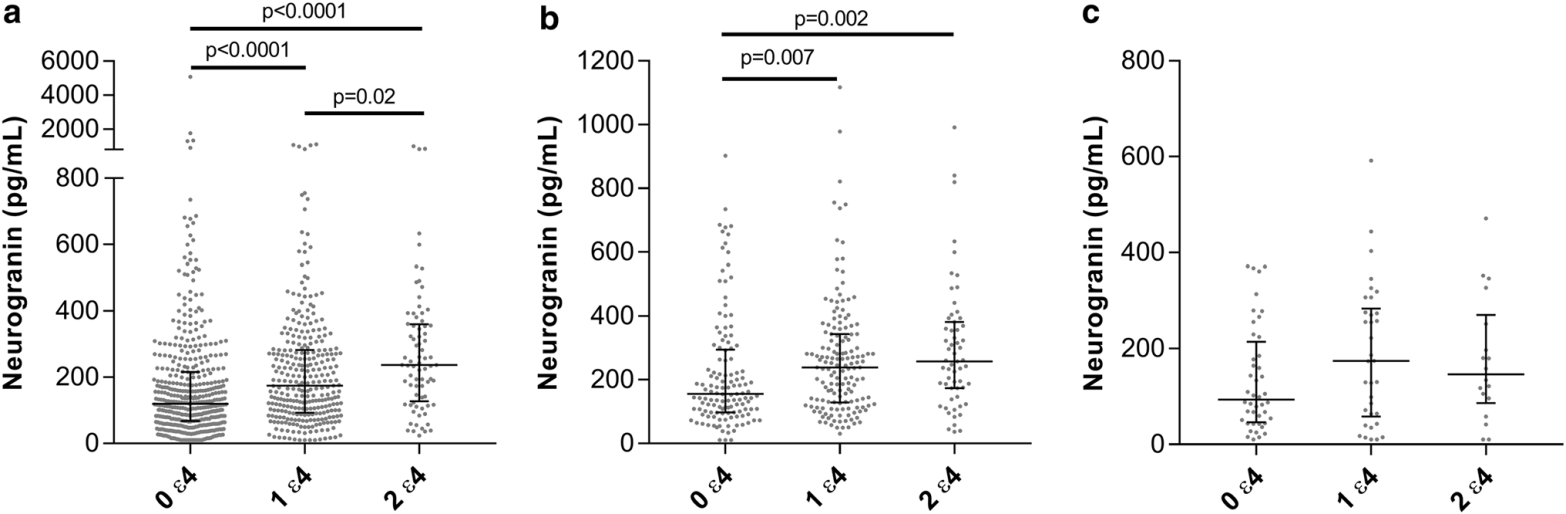
Scatterplots displaying the CSF Ng concentrations in patients having zero, one or two *APOE ε4* alleles in **a** all subjects included in the study **b** clinically diagnosed ADD and **c** clinically diagnosed MCI. The bars presented in the figures are medians with interquartile ranges and comparisons between groups were performed using Kruskal–Wallis test, followed by the Mann–Whitney *U* test

When grouping according to the number of *APOE* ε4 alleles, CSF Ng increased in a gene dose-dependent manner with the highest Ng concentrations in the group homozygous for the ε4 allele (Fig. 5a). CSF Ng concentrations were also strongly associated with the number of copies of the ε4 allele within the ADD group with the highest concentrations in the group having two copies of the ε4 allele (*p* = 0.002) (Fig. 5b). There was a trend towards increased CSF Ng concentrations with increasing number of *APOE* ε4 alleles within the MCI group, but this did not reach statistical significance (Fig. 5c).

### CSF Ng in relation to autopsy-confirmed patients

**Fig. 6:**
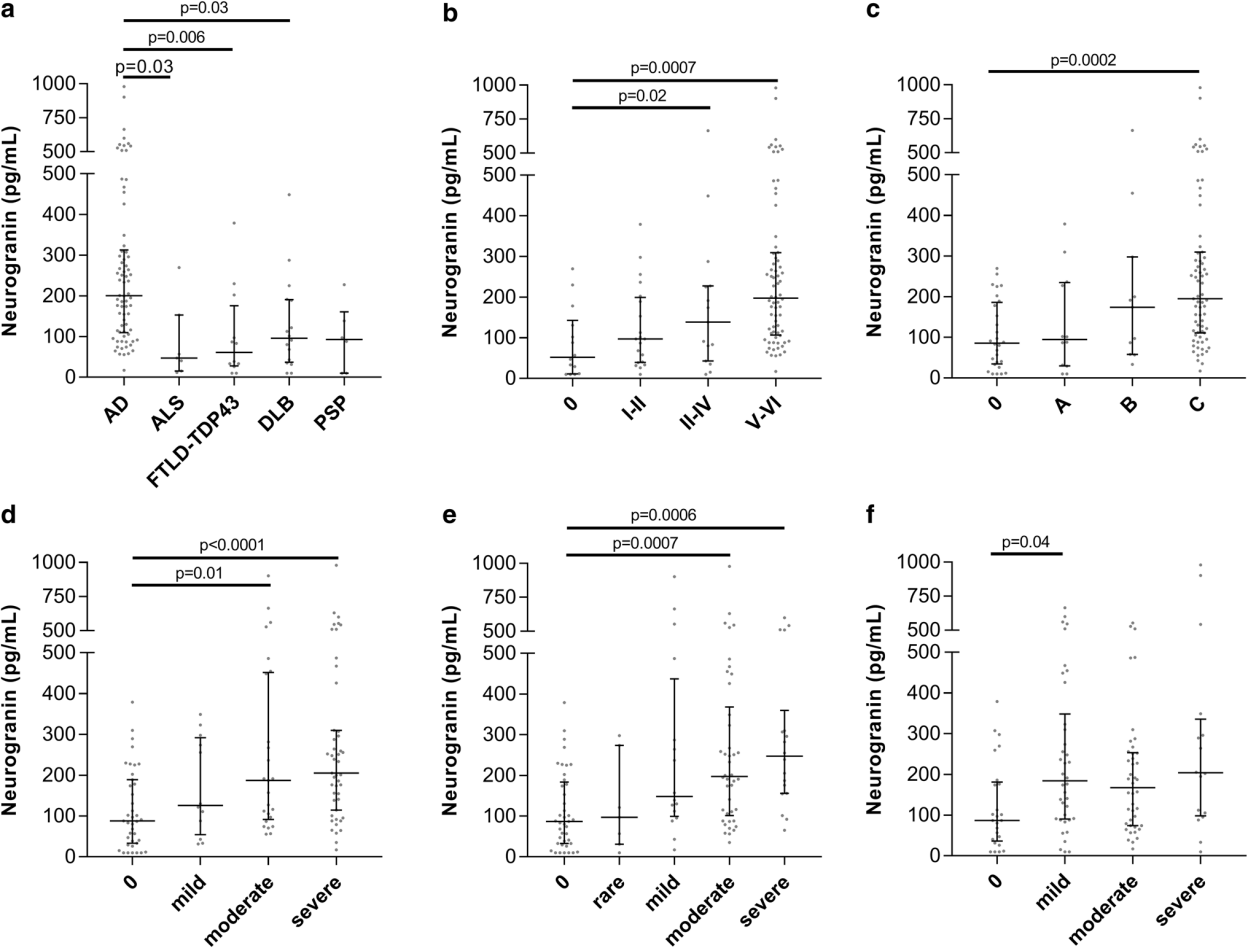
Scatterplots displaying the CSF Ng concentrations in relation to **a** autopsy confirmed cases, **b** tau neurofibrillary tangles, **c** Aβ neuritic plaques, **d** Aβ neuritic plaques in amygdala, **e** Aβ neuritic plaques in hippocampus, and **f** neuronal loss in hippocampus. The bars presented in the figures are medians with interquartile ranges and comparisons between groups were performed using Kruskal–Wallis test, followed by the Mann–Whitney *U* test

In the autopsy-confirmed cases with definitive diagnoses, CSF Ng was significantly increased in ADD compared to DLB (*p* = 0.03), frontotemporal lobar degeneration FTLD (*p* = 0.006), and ALS (*p* = 0.03) (Fig. 6a). We also examined the association between CSF Ng and the burden of neurofibrillary tangles and plaques in the brain. Braak neurofibrillary staging and CERAD were used to classify the neuropathology into four groups as described previously [37] ranging from no AD to high-level AD pathology. In this analysis, CSF Ng was significantly increased in the high-level pathology AD group having most widespread tau (*p* = 0.0007) and Aβ plaque (*p* = 0.0002) pathology compared to no (or negligible) AD pathology (Fig. 6b, c).

Since Ng is highly expressed in the cerebral cortex, hippocampus and amygdala, which are the same brain regions that are affected in ADD [42], we investigated the relationship between CSF Ng concentrations and neuropathology findings in amygdala and hippocampus. While there were no associations between CSF Ng and tau neurofibrillary tangles, α-synuclein, or TDP-43 load in amygdala or hippocampus, we found that higher Aβ plaque load in the amygdala and hippocampus correlated with increasing CSF Ng concentrations (*p* < 0.0001 in amygdala and *p* = 0.0006 in hippocampus) (Fig. 6d, e). In addition, we also found an association between CSF Ng concentrations and neuronal loss in the hippocampus (*p* = 0.04) (Fig. 6f) while there were no association between CSF Ng and neuronal loss in the amygdala.

**Fig. 7:**
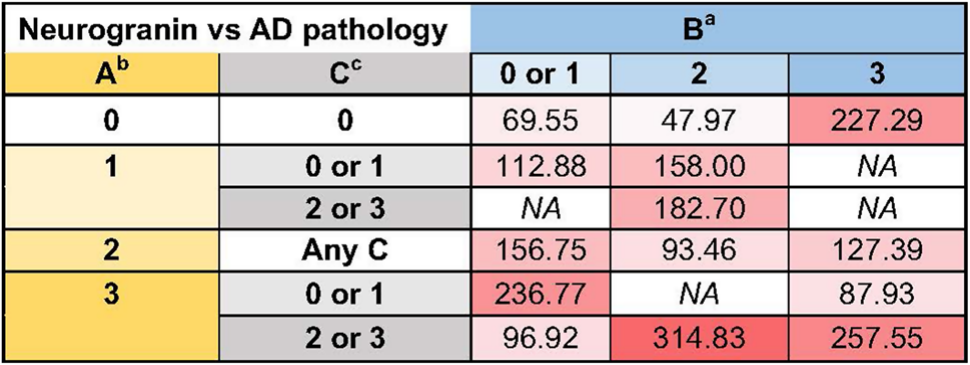
(a) A heat map displaying the mean CSF Ng concentrations for 114 autopsy-confirmed subjects as defined by the ABC classification scheme. The ABC score incorporates histopathologic assessments of Aβ deposits (A), staging of neurofibrillary tangles (B), and scoring of neuritic plaques (C). ^a^ NFT stage should be determined by the method of Braak [5, 6], ^b^ Aβ/amyloid plaque score should be determined by the method of Thal et al. [49], ^c^ Neuritic plaque score should be determined by the method of CERAD [35]

Next we averaged CSF Ng values for subjects with postmortem evaluation of AD pathology according to a pathology classification table as previously described [37]. We then aggregated CSF Ng values over each pathology stage for each of the three classification schemes to understand the relationship between CSF Ng and each classification scheme individually. We found a significant difference in CSF Ng concentrations between the different pathology stages in the Thal stage (*H* = 22.75, *p* < 0.0001), as well as in the Braak stage (*H* = 27.32, *p* < 0.0001), and in the CERAD score (*H* = 25.50, *p* < 0.0001) (Fig. 7). After post hoc analysis, there was a significant difference in CSF Ng between stages 0 and 3 (*p* = 0.0003) and between stages 1 and 3 (*p* = 0.02) in Thal staging, along with a significant difference between stages 2 and 3 which was no longer significant after multiple comparisons correction (*p* = 0.03 before, *p* = 0.12 after). For Braak staging, there was a significant difference in CSF Ng between stages 0 and 3 (*p* < 0.0001) and between stages 1 and 3 (*p* = 0.002), along with a significant difference between stages 0 and 2 which was no longer significant after multiple comparisons correction (*p* = 0.04 before, *p* = 0.18 after). Finally, in the CERAD scheme there was a significant difference in CSF Ng between stages 0 and 3 (*p* < 0.0001) and a nearly significant difference between stages 1 and 3 (*p* = 0.054).

## Discussion

Here, we report on CSF Ng concentrations in both clinically diagnosed and in neuropathologically confirmed subjects, and show that CSF Ng concentrations were significantly higher in ADD compared to DLB, FTD and ALS. Further, increased CSF Ng concentrations correlated with increased Aβ plaque load, specifically in the hippocampus and amygdala, probably reflecting synaptic damage induced by aggregation of Aβ and accumulation in plaques.

CSF Ng is a well-replicated biomarker for ADD (http://www.alzforum.org/alzbiomarker). As Ng is expressed in dendrites and as CSF Ng concentrations correlate with memory impairment and reduced cerebral glucose metabolism in ADD-affected brain regions [39], this biomarker has been proposed to reflect synaptic dysfunction in ADD. Importantly, CSF Ng appears to be an ADD-specific biomarker; its concentration is unaltered or even reduced in several neurodegenerative diseases, including FTD, PD and atypical parkinsonian disorders [25, 41, 53]. Here, we re-examine the ADD specificity of CSF Ng in a large cross-sectional serie of neurodegenerative diseases. We extended the analysis by examining the marker in pathologically confirmed cases and assessed its correlation with several pathological changes in the brain.

A common feature in several neurodegenerative diseases affecting memory is the presence of brain amyloidosis, including plaques (Aβ), tangles (tau) and Lewy bodies (α-synuclein), and neuropathological studies have shown that ADD patients often have other concomitant pathologies, besides plaques and tangles [12, 24, 37]. We found increased CSF Ng concentrations with increased Thal, CERAD and Braak scores demonstrating that Ng is linked to the ADD pathology. Our results also suggest that CSF Ng values began to differentiate relatively earlier in Braak staging (between stages 0 and 2) compared to the others, while CSF Ng differentiation between stages persisted relatively late in Thal staging (between stages 2 and 3). CSF Ng concentrations differentiated least in CERAD scores compared to the others. We also show that increased plaque load in the hippocampus and amygdala was paralleled by increased CSF Ng concentrations while there was no association between tau, α-synuclein or TDP-43 pathology and CSF Ng. These data support the hypothesis that Aβ neurotoxicity specifically affects synaptic function, and that plaque pathology associated with cognition can be translated into high CSF Ng concentrations. In addition, we found a weak negative correlation between CSF Aβ and CSF Ng concentrations in the ADD group. These data further support the association between CSF Ng and plaque load. The deposition of Aβ into plaques leads to a lowering of CSF Aβ42 concentrations, while CSF Ng is believed to increase as a consequence of synaptic dysfunction and degeneration. There was also an association between neuronal loss in hippocampus and CSF Ng in the groups with negligible ADD neuropathology and low-level ADD which may reflect that the neuronal loss at early stages is most dynamic. However, this needs to be further evaluated and confirmed in additional studies.

We found that CSF Ng concentrations in ADD were higher in patients having one or two copies of the *APOE* ε4 allele compared to non-carriers. The same trend was observed for the MCI group but there was no statistically significant difference between the groups, which may be due to low number of patients included. This is in agreement with a previous study and shows that the number of copies of the ε4 allele may be associated with synaptic dysfunction which is reflected in the CSF as higher Ng concentrations [47]. One explanation could be that ADD patients with two copies of the ε4 allele have an increased plaque load compared to patients with no ε4 allele [11]. Thus, the number of ε4 allele copies affects the amount of plaques (and/or neurotoxic Aβ species) which in turn induces synaptic pathology.

There was a significant association between the CSF Ng concentration and rate of cognitive decline, as measured by drop in MMSE scores per year in the MCI group. This is in agreement with previous studies [27] again showing that Ng is linked to synaptic function and cognition at early stages.

We show that increased CSF Ng concentration occurs only in ADD and not in the other neurodegenerative disorders investigated in this study. One exception was lvPPA which displayed similar CSF Ng concentrations as those found in ADD. A plausible explanation is that a large number of lvPPA patients actually have ADD pathology [20, 33]. However, there were no correlations between CSF p-tau, t-tau, or Aβ42 with CSF Ng in the lvPPA group. Future studies should investigate pathology-confirmed lvPPA cases with regional plaque distribution to establish if this finding still holds.

The CSF Ng concentrations were significantly increased in ADD compared to svPPA and nfvPNFA but also increased in lvPPA compared to bvFTD. This may be explanted by that svPPA and nfvPNFA are more predictive of FTLD-TDP and FTLD-tau, respectively. Thus, the finding reflects that lvPPA patients are likely atypical variants of ADD [13]. In addition, out of the 12 lvPPA patients included in the study, ten had available CSF Aβ42 and t-tau concentrations measurements and eight of these were ADD biomarker positive, further supporting this statement.

bvFTD and PPA are diagnosed according the appearance of the symptoms. In the present study, CSF Ng concentrations in bvFTD were significantly lower than in ADD and lvPPA but also lower compared to CTRL which is in agreement with a previous study [53]. In addition, there were no correlations between CSF Ng and the core CSF ADD biomarkers in bvFTD. However, adding CSF Ng to the core AD biomarkers (Aβ, t-tau and p-tau) significantly increased the accuracy in distinguishing between CTRL and bvFTD and MCI vs bvFTD. Thus, adding CSF Ng to the core AD biomarkers (Aβ, t-tau and p-tau) adds diagnostic information. Further studies are warranted to confirm these findings since the pathological heterogeneity of bvFTD may contribute to the results [22].

Interestingly, there was a strong positive correlation between CSF Aβ42 and Ng levels in the ALS group and at the same time, CSF Ng concentrations were significantly lower in ALS compared to ADD and even slightly lower than in CTRL. It is known that a small percentage of ALS patients have ADD pathology [7] and it was shown recently that both amyloid precursor protein (APP) and intracellular Aβ are overexpressed in the hippocampus in ALS compared to CTRL [14]. In animal models of ADD it has been shown that increasing the soluble APP fragment cleaved by α-secretase may improve cognition and rescue long-term potentiation (LTP) [34, 38]. Ng has also been shown to be associated with cognition and to play a role in LTP [18, 36, 54]. In addition, there was no correlation between neuritic plaque load and the CSF Ng concentrations in ALS. Thus, it is tempting to speculate that APP is involved in cell survival in ALS and that increased APP and Aβ expression in ALS, without plaque pathology, is reflected as low concentrations of CSF Ng which in turn reflect prosperous synapses. Further studies are warranted to confirm these findings.

When evaluating novel CSF biomarkers for ADD, it is important to consider that concomitant pathologies are common, especially in elderly patients with dementia. To test CSF Ng as a specific biomarker for ADD, we investigated its performance in ADD and other neurodegenerative disease cases with definitive diagnoses determined by postmortem examination. However, the number of patients was low in some groups. Thus, larger studies including neuropathology confirmed patients are warranted.

The study has a number of limitations that should be acknowledged. First, the sample size in some subgroups was small and the risk of false-positive statistical test warrants replication of the most interesting findings. Therefore, interpretation should be made with caution. Second, although our study replicates the specificity of CSF Ng for ADD, a number of novel findings regarding what CSF Ng may add to other markers need to be replicated in independent cohorts. Thus, in the future, we will seek verification of these results in other, independent datasets to investigate their clinical relevance.

Some of the highest CSF Ng levels were found in two individuals in the CTRL group. Data from neuropathological examination were not available and, therefore, it is unknown whether these high levels are due to that these individuals have AD, or possibly other forms of pathology, a change in the overall clearance and production of Ng, or possibly if the very high values have analytical or technical explanations.

In conclusion, CSF Ng is a biomarker specifically reflecting synaptic pathology in ADD and its concentration is linked to the extent of plaque pathology in the hippocampus and amygdala. The findings support the use of CSF Ng as a biomarker for diagnosing ADD and also for treatment trials, where disease-modifying drugs are evaluated, to monitor if treatment restores synaptic function in the patients.

## Electronic supplementary material

Below is the link to the electronic supplementary material.

Supplementary material 1 (XLSX 11 kb)

Supplementary material 2 (PDF 61 kb)

Supplementary material 3 (XLSX 10 kb)
