# Supplementary material for: Cerebrospinal fluid neurogranin concentration in neurodegeneration: relation to clinical phenotypes and neuropathology
Source: Acta Neuropathol. 2018 Apr 26;136(3):363–76. doi: 10.1007/s00401-018-1851-x (PMC6096740; doi:10.1007/s00401-018-1851-x)
Supplement: Supplementary file 2 — Supplementary material 2 (PDF 61 kb) [file 401_2018_1851_MOESM2_ESM.pdf]

Online Resource 2. Statistics

We developed a biomarker index model with the overarching goal of accurately discriminating between two disease groups on the basis of their CSF biomarker measurements. The baseline model included CSF Aβ, CSF t-tau and CSF p-tau, and the Ng model included CSF Ng in addition to the three aforementioned CSF measurements.

The discriminator itself is a support vector machine (SVM), which is a well-established and readily interpretable classification model commonly used in the medical literature to classify subjects between two groups. We initially tried a simple logistic regression model as our discriminator under the principle of Occam's Razor, but found empirically that SVM readily outperformed logistic regression in nearly every task.

To gather information on biomarker index, we ran our model using repeated stratified 5-fold cross validation. This means that we split the entire dataset into five groups in which each of the five groups had an equal class distribution (e.g. the same percentage of AD subjects were in each group) and then performed traditional cross validation on those five groups. Because cross validation accuracy with datasets of this size is highly sensitive to random group membership, we then repeated this cross validation procedure 20 times to obtain a sample population of accuracy scores.

For a given two-group classification task (e.g. ADD vs CTRL), we repeated the above steps for both the baseline (Aβ, t-tau, p-tau) and Ng (Ng, Aβ, t-tau, p-tau) models, resulting in two sets of 20 accuracy scores. From there, we calculated the relative increase (or decrease) in classification accuracy obtained by including Ng in the baseline model and performed a two-sided student's t-test for difference of means between the 20 classification scores to test whether including Ng significantly increased (or decreased) the classification accuracy of the biomarker index model.

Finally, we repeated this above procedure for each possible two-pairs of diseases represented in our dataset to obtain a matrix of scores representing the contribution and significance of Ng being included in the biomarker index model.

Our justification for this relatively straight-forward procedure was to understand whether including Ng in a biomarker index model contributed any additional information in the context of discriminating between neurodegenerative disorders, and to understand between which two disorders specifically Ng was useful in discriminating.

"Cerebrospinal fluid neurogranin concentration in neurodegeneration – relation to clinical phenotypes and neuropathology", Acta Neuropathologica, Portelius et al., correspondence: Erik Portelius, Institute of Neuroscience and Physiology, Department of Psychiatry and Neurochemistry, the Sahlgrenska Academy at the University of Gothenburg, Mölndal, Sweden, Phone: +46 31 343 23 90, e-mail: erik.portelius@neuro.gu.se
